# Traumatic knee injuries and career drop-outs in adolescent competitive alpine skiers aged 15–19: a longitudinal 4-year follow-up study examining rates, biomechanical injury risk factors and potential reasons for quitting

**DOI:** 10.1080/07853890.2025.2532118

**Published:** 2025-07-15

**Authors:** Jonas Hanimann, Daniel P. Fitze, Thomas Koller, Flavia Schürmann, Stefan Fröhlich, Georg C. Feuerriegel, Christoph Stern, Johannes Scherr, Eling D. de Bruin, Reto Sutter, Jörg Spörri

**Affiliations:** a Sports Medical Research Group, Department of Orthopaedics, Balgrist University Hospital, University of Zurich, Zurich, Switzerland; b University Centre for Prevention and Sports Medicine, Department of Orthopaedics, Balgrist University Hospital, University of Zurich, Zurich, Switzerland; c Department of Health Sciences and Technology, ETH Zürich, Zurich, Switzerland; d Department of Radiology, Balgrist University Hospital, University of Zurich, Zurich, Switzerland; e Department of Health, OST – Eastern Switzerland University of Applied Sciences, St. Gallen, Switzerland

**Keywords:** Injury prevention, skiing, athletes, adolescence, knee injuries

## Abstract

**Introduction:**

The aims of this longitudinal 4-year follow-up study were (1) to assess traumatic knee injuries and career drop-out rates in competitive alpine skiers from mid- to late adolescence (i.e. 15–19 years), (2) to investigate the magnetic resonance imaging (MRI) abnormalities in the knees of skiers reaching late adolescence, and (3) to evaluate the biomechanical landing patterns of mid adolescent skiers and to compare skiers who experienced an anterior cruciate ligament (ACL) injury during the 4-years follow-up and those who did not.

**Materials and methods:**

At baseline, knee flexion angle and medial knee displacement during drop jump landing were biomechanically assessed in 108 skiers aged 14.83 ± 0.58 years. During a 4-year longitudinal follow-up, 45 skiers dropped out of the study owing to lack of interest. This left a remaining cohort of 63 skiers. After 4 years, 63 skiers (aged 18.95 ± 0.64 years) were retrospectively interviewed about their knee injuries that had occurred over the past 4 years. They were also asked whether they had quit their sports careers since then and, if so, when and why. Additionally, their knee joints were imaged with MRI.

**Results:**

During the 4-year follow-up, 21 skiers in the cohort of 63 skiers experienced traumatic knee injuries, 9 of whom experienced ACL injuries. Seven ACL injuries occurred in female skiers, and 2 occurred in male skiers (*p* = 0.044). Thirty-nine skiers (61.9%) ended their sport careers, 41.0% for health-related reasons. At baseline, female skiers who experienced ACL injury during follow-up presented smaller knee flexion angles during drop jump landing than non-injured skiers did (*p* = 0.013).

**Conclusion:**

Between 15 and 19 years of age, one-third of competitive alpine skiers suffer from traumatic knee injury and more than half of skiers end their sports careers. In female skiers, stiff landing strategies may be considered an important but modifiable risk factor that could be targeted through systematic instructions and training.

## KEY MESSAGES

- In the 4 years between 15–19 years of age, one-third of competitive alpine skiers suffer from traumatic knee injury, with anterior cruciate ligament (ACL) injuries being the most prevalent type of injury. Compared with male skiers, female competitive alpine skiers are more likely to suffer ACL injuries during adolescence.
- More than half of skiers end their sports careers during mid to late adolescence, with health-related reasons serving as one of the main explanations.
- ‘Stiff landing’ strategies may favour ACL injuries in female skiers.

## Introduction

Competitive alpine skiing carries a high risk of injury [1–9]. Knee injuries, particularly anterior cruciate ligament (ACL) injuries, are among the most frequent injuries in competitive alpine skiing [4–9]. ACL injuries are often accompanied by concomitant injuries, such as meniscus tears or injuries to the collateral ligaments [10–12]. The short-term consequences of such injuries are typically surgery and/or long absences from sports participation [8]. Moreover, in the long term, re-injury rates are high [13,14], and the risk for osteoarthritis is increased [15]. Accordingly, knee injuries (and especially ACL injuries) represent a major burden for athletes participating in competitive alpine skiing.

While traumatic knee injury rates in competitive alpine skiers are relatively low before the growth spurt (23.2% over a two-year period) [16], they increase in the years surrounding the growth spurt (21.3% over a one-year period) [17], and reach rates comparable to those at the elite level in adolescent skiers after the growth spurt (approximately 20% over a one-year period) [4,6]. Recent studies reported a high prevalence of knee overuse injuries in youth skiers aged approximately 15 years [17,18]. In these studies, overuse injuries were dominant in early adolescence; however, they partially decreased with increasing age during adolescence, whereas traumatic injuries became more common [6,19–22].

In elite competitive alpine skiers, the accumulated risk of ACL injury as the most frequent type of traumatic knee injuries is between 5% and 15% [1,4–6]. However, longitudinal data on traumatic knee injuries and ACL injuries in a cohort of athletes followed throughout adolescence are widely lacking. In addition to substantial physical changes, adolescence is a highly challenging phase in the sports careers and lives of athletes in terms of both sports performance and personal development. Many athletes quit their sports careers during this phase because of increasing demands in education, a general shift in interest, and increasing sports-related health problems. The exact drop-out rates in adolescent competitive alpine skiers and the associated reasons are largely unknown. However, profound understanding could contribute to the sports losing fewer athletes.

In competitive alpine skiing, injury mechanisms leading to ACL injuries typically include aggressive quadriceps loading that induces anterior tibial translation relative to the femur while jump landing [23–25], or a combination of dynamic knee valgus with aggressive quadriceps action inducing additional tibial internal rotation [23,26,27]. In various sports, it has been reported that jump landing strategies with less flexed hips and knees (i.e. stiff landings) and with increased dynamic knee valgus are likely more common in athletes who are prone to ACL injuries, especially in female athletes [28–33]. Moreover, simulation studies of ACL injury-prone landing manoeuvres in competitive alpine skiing demonstrate that neuromuscular control patterns characterised by increased hip and knee flexion (i.e. less stiff landings) reduce the peak strain acting on the ACL [24,25]. Studies examining differences in the landing patterns of skiers at baseline in relation to the occurrence of ACL injuries during longitudinal follow-up could provide real-life information on this topic; however, these studies have yet to be conducted.

The aims of this study were (1) to assess traumatic knee injuries and career drop-out rates in competitive alpine skiers over a period of 4 years from mid- to late adolescence (i.e. 15–19 years), (2) to investigate the magnetic resonance imaging (MRI) abnormalities in the knees of skiers reaching late adolescence (i.e. at the age of 19 years), and (3) to evaluate the biomechanical differences in landing patterns at baseline between skiers who experienced an anterior cruciate ligament (ACL) injury during the 4-years follow-up and those who did not. It was hypothesised that (i) there are a large number of skiers who experience traumatic knee injuries and quit their sport careers at this particular age, primarily for health reasons, (ii) by late adolescence there is a significant amount of trauma-related MRI abnormality in the knees of skiers, and (iii) mid adolescence there are distinct patterns in the jumping landing biomechanics of skiers who experience an ACL injury in the subsequent 4 years compared to those who do not.

## Materials and methods

### Participants and study design

This study was designed as a longitudinal study with cross-sectional, prospective, retrospective components.

At the cross-sectional biomechanical baseline assessment, all of the 108 participants aged 14.83 ± 0.58 years were competitive alpine skiers and belonged to a certified regional performance centre in Switzerland. This cohort is the same as that of another study [18]. During the 4-year longitudinal follow-up period, 45 skiers dropped out of the study owing to lack of interest. This left a remaining cohort of 63 skiers. Accordingly, after 4 years, 63 skiers (aged 18.95 ± 0.64 years) were retrospectively interviewed about their knee injuries that had occurred over the past 4 years. Moreover, they were also asked whether they had quit their sports careers since then and, if so, when and why. Additionally, their knee joints were cross-sectionally imaged with MRI.

The analyses of the present study are based on a sample of 63 competitive alpine skiers (29 females and 34 males). The inclusion criterion was data availability from both baseline and follow-up assessment. None of the potential participants were excluded on the basis of the exclusion criteria, namely, those diagnosed with (or not yet fully recovered from) an acute knee sprain at the time of the study or those with systemic diseases such as inflammatory arthritis and diabetes mellitus that affect joint health and/or tendon properties. The current study protocol was approved by the responsible cantonal ethics committee (KEK-ZH-NR: 2017-01395). At both assessment timepoints, all participants were informed about the study in detail and signed a written informed consent form. For participants younger than 14 years of age, written informed consent was provided by the participants’ legal guardians/next of kin. The data are presented in line with the STROBE Statement [34].

### Data collection

#### Biomechanical assessments at baseline

As part of the baseline assessment, the knee flexion angle and medial knee displacement (as defined below) were measured during drop jump landing at the time of the maximal ground reaction force. For the underlying biomechanical assessment, a three-dimensional motion capture system consisting of 8 cameras (Vicon, Oxford Metrics) and two force plates (SP Sportdiagnosegeräte GmbH) was used. Both systems run synchronously at 200 Hz and 2000 Hz. Vicon Nexus software and a modified Plug-In Gait model were used for marker and trajectory detection (Vicon Nexus v2.6, Oxford Metrics) [35]. The participants were equipped with 31 reflective skin markers [35]. A static calibration was subsequently conducted; therefore, 4 additional markers were used at both the medial femur epicondyles and the medial malleoli. For the assessment of drop jump landings, participants were advised to drop off a 32 cm high box in an upright position, land with their feet on separate, adjacent force plates, and perform a maximal high vertical jump while the ground contact time was kept as short as possible. Trials were repeated if participants (i) did not land on the force plates, (ii) lost hand-hip contact, (iii) actively jumped from the box instead of dropping, or (iv) hesitated to land before performing the maximal vertical jump. Two valid trials were prescribed, and participants continued performing trials with a minimal recovery time of 15 s until two valid trials were recorded.

For post-processing, the data were transferred to MATLAB (MATLAB R2016b, The MathWorks, Inc.), and customized scripts were used. Marker trajectories with gaps were interpolated up to a length of 0.05 s. A Butterworth low-pass filter was used for analogue force plate data (cut-off frequency of 200 Hz). Medial knee displacement was defined as the rectangular distance of the knee joint from the reference plane, which was set to one frame before ground contact. The reference plane consisted of the hip, knee, and ankle joint centres, and ground contact was considered to exceed the 25 N threshold at the force plates. The knee flexion angle was defined as the angle between hip, knee, and ankle joint centres. Both the maximal knee flexion angle and the medial knee displacement at the maximal ground reaction force were averaged over both landings and for both legs.

#### Magnetic resonance imaging follow-up assessment

At the follow-up assessment, magnetic resonance (MR) imaging of the skiers’ knee joints was conducted. Both knees of all skiers were scanned in a 3 T magnetic resonance (MR) scanner (MAGNETOM Prisma, Siemens Healthcare, Erlangen, Germany) with a dedicated knee coil. For image collection, a standardised isotropic fat-suppressed T2-weighted three-dimensional sequence (SPACE) was acquired with the following parameters: in-plane resolution 0.63 × 0.63 mm, slice thickness 0.63 mm, field of view (FOV) 160 × 160, matrix 256 × 256, echo time (TE) 108 ms, repetition time (TR) 1000 ms, receiver bandwidth 415 Hz/Px, parallel imaging acceleration factor, 4; and duration, 4:42 min. Image analysis was performed by an experienced radiologist specialising in musculoskeletal imaging.

#### Retrospective 4-year follow-up interviews

All skiers were retrospectively interviewed *via* a 15-minute Microsoft Teams call to collect information on traumatic knee injuries that occurred during the 4-year period between the baseline and follow-up assessments. A traumatic injury was defined as an injury with a single identifiable event that resulted in a sudden onset of symptoms (i.e. any complaint regardless of the need for medical attention) [36]. Moreover, every injury was also categorized by body location, type, and severity, as defined by Fuller and colleagues [36]. If a single event resulted in multiple injuries, each was listed separately, except if the injuries were in the exact same body part, e.g. ACL injury and meniscus tear. For the subcategory of ACL injuries, a specific medical diagnosis had to be available. Additionally, the skiers’ status regarding their current sports participation at the time of the follow-up assessment was assessed and classified as follows: active, retired, and, if retired, skiers were asked about their reasons for quitting their sport.

### Statistical analysis

Statistical analysis was performed with IBM SPSS software version 28 (IBM, Armonk, United States). The figure was created with R (version: 2023.09.1 + 494). Shapiro–Wilk tests were used to check for a normal distribution, and all parameters except *delta age* and *delta height* were normally distributed. Unpaired sample t tests were used to assess sex differences in baseline characteristics; for *delta age* and *delta height*, Mann–Whitney U tests were used. The occurrence of trauma-related knee MR imaging abnormalities was described as the number of skiers with corresponding indications, as well as their percentage proportion (number of subjects affected/total number of subjects per group or subgroup * 100). Corresponding sex differences were analysed using Pearson Chi^2^ tests. Traumatic knee injuries in the 4 years between baseline and follow-up drop-out rates were reported as absolute numbers of skiers and related percentages. The sex differences in acute knee injuries were analysed by Pearson Chi^2^ tests; for some injuries with expected cell counts smaller than 5, Fisher’s exact test was used. To assess the differences in landing patterns at baseline between skiers who experienced ACL injury during follow-up and those who did not, unpaired sample *t*-tests were used. For all statistical tests, the significance level was set at *p* < 0.05.

## Results

### Baseline and follow-up characteristics of the study cohort

The baseline and follow-up characteristics of the study cohort are presented in Table 1. At the follow-up, male skiers were taller (*p* < 0.001) and heavier (*p* < 0.001) than female skiers were. Moreover, male skiers gained substantially more weight (*p* < 0.001) and height (*p* < 0.001) during the 4-year follow-up period.

**Table 1.** Mean values of the baseline and follow-up characteristics of female and male skiers, including height, weight, delta height, and Delta weight.

	Baseline				Follow-up				Delta time		
	Female (*n* = 29)	Male (*n* = 34)	Delta sex	*p*-value	Female (*n* = 29)	Male (*n* = 34)	Delta sex	*p*-value	Female (*n* = 29)	Male (*n* = 34)	*p*-value
Age [years]	14.83	14.87	0.04	0.803	18.92	18.97	0.05	0.779	4.09	4.09	0.820
Height [cm]	163.61	169.64	6.03	<0.001	166.59	177.90	11.31	<0.001	2.98	8.26	<0.001
Weight [kg]	55.29	59.50	4.21	0.058	62.43	76.56	14.13	<0.001	7.14	17.06	<0.001

### Traumatic knee injuries in the 4 years between baseline and follow-up

**Figure 1.**
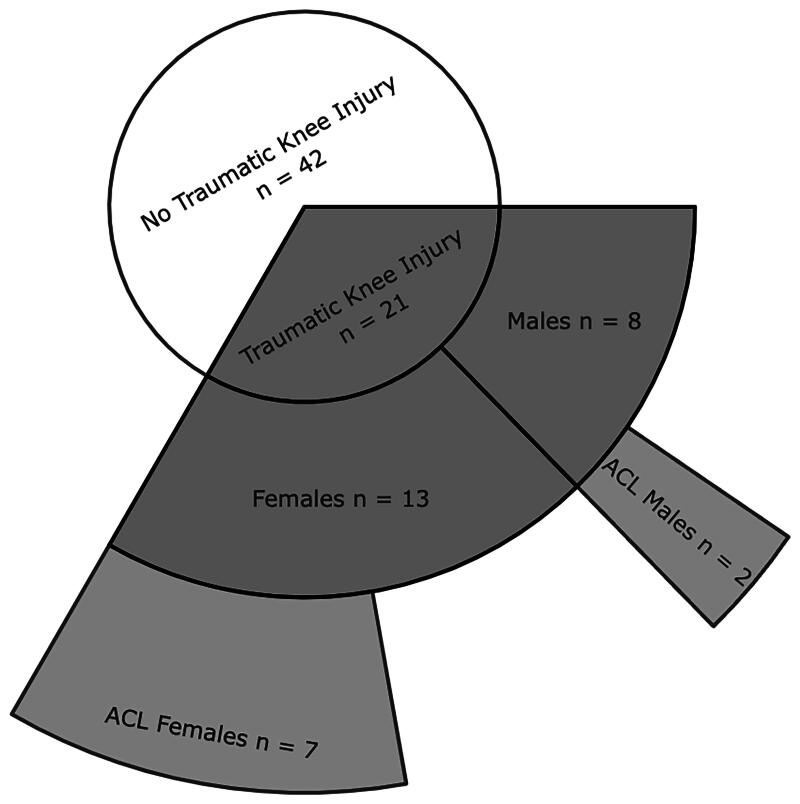
Graphical visualization of the data from Table 4: prevalence of traumatic injuries and ACL injuries during the 4-year follow-up.

During the four-year period between baseline and follow-up, 21 skiers experienced traumatic knee injuries, 9 of whom experienced ACL injuries (Table 2 and Figure 1). All ACL injuries occurred in combination with collateral ligament and/or meniscus injuries. One female skier had two ACL injuries, one on each side, from two separate events. Another female skier experienced both an ACL injury and an ACL re-injury on the same side during the 4-year follow-up period. One male skier who suffered an ACL injury before the baseline assessment experienced re-injury during the follow-up assessment. As shown in Table 2, there was no significant difference in the distribution of traumatic knee injuries between male and female skiers (χ^2^(1) = 3.195, *p* = 0.074); however, there were significantly more ACL injuries in female skiers than in male skiers (χ^2^(1) = 4.260, *p* = 0.044).

**Table 2.** Prevalence of traumatic injuries and ACL injuries with absolute numbers and percentage values (in brackets).

	Total (*n* = 63)	Females (*n* = 29)	Males (*n* = 34)	Chi^2^	*p* value
Traumatic Injury during 4 years follow-up	21 (33.3%)	13 (44.8%)	8 (23.5%)	3.195	0.074
Skiers with ACL Injuries during 4 years follow-up	9 (14.3%)	7 (24.1%)	2 (5.9%)	4.260	0.044^#^

Significant differences in the distributions between sexes were determined *via* pearson’s Chi^2^ tests (*p* < 0.05). ^#^Fisher’s exact test (*p* < 0.05).

### Career status at follow-up

Among the 63 skiers, 39 skiers (61.9%) stopped competitive skiing during the 4-year period after baseline, and 24 were still active (Table 3). Eighteen male skiers and 21 female skiers ended their careers, whereas 16 and 8 were still active, respectively. As shown in Table 3, 23 of the 39 skiers who stopped competitive skiing (59.0%) did so for non-health-related reasons (14 males and 9 females). The other 16 skiers (41.0%) ended their sports careers for health-related reasons, and 9 (23.1%) quit because of a combination of health-related and other reasons.

**Table 3.** Presentation of the skiers who were still active in skiing at the end of the 4-year observation period, the skiers who ended their career within the 4-year observation period, and the reasons for quitting.

	Total	Females	Males
**Still active**	24 (38.1%)	8 (27.6%)	16 (47.1%)
**Not active**	39 (61.9%)	21 (72.4%)	18 (52.9%)
Health-related reasons	7 (17.9%)	5 (23.8%)	2 (11.1%)
Non-health-related reasons	23 (59.0%)	9 (42.9%)	14 (77.8%)
Health-related reasons AND non-health-related reasons	9 (23.1%)	7 (33.3%)	2 (11.1%)

### Trauma-related MR imaging abnormalities in the knee at the follow-up

All trauma-related MR imaging abnormalities in the knee that were detected *via* MR imaging at the follow-up assessment are presented in Table 4. Given that these findings are cross-sectional, it is not differentiated if they originated during the 4-year follow-up period. At follow-up, thirty-nine skiers (61.9%) had at least one trauma-related MR imaging abnormality in the knee (in females: 18 skiers (62.1%); in males: 21 skiers (61.8%)). There was no sex difference in the percentage proportion of skiers who presented with at least one trauma-related MR imaging abnormality. The highest prevalence was found for cartilage lesions, followed by bone marrow edema and ACL and PCL injuries. Sex differences were found for ACL and PCL injuries (χ^2^(1) = 6.292, *p* = 0.012).

**Table 4.** Prevalence of trauma-related MR imaging abnormalities with absolute numbers and percentage values (in brackets).

MR imaging abnormality	Overall (*n* = 63)	Female (*n* = 29)	Male (*n* = 34)	Chi^2^	*p* value
≥ 1 trauma-related MR Imaging Abnormality	39 (61.9%)	18 (62.1%)	21 (61.8%)	0.001	0.980
ACL and PCL Injuries	13 (20.6%)	10 (34.5%)	3 (8.8%)	6.292	0.012
Meniscal Alterations	11 (17.5%)	5 (17.2%)	6 (17.6%)	0.002	0.966
Meniscal Tear	10 (15.9%)	6 (20.7%)	4 (11.8%)	0.934	0.492^#^
Cartilage Lesion	14 (22.2%)	9 (31.0%)	5 (14.7%)	2.414	0.120
Joint Effusion	6 (9.5%)	3 (10.3%)	3 (8.8%)	0.042	1.000^#^
Bone Marrow Edema	13 (20.6%)	3 (10.3%)	10 (29.4%)	3.474	0.062
Collateral Ligament Abnormalities	3 (4.8%)	3 (10.3%)	0 (0.0%)	3.693	0.092^#^

Significant differences in distribution between sexes were determined via pearson’s Chi^2^ tests (*p* < 0.05). ^#^Fisher’s exact test (*p* < 0.05).

### Baseline biomechanical measures of skiers that suffered an ACL injury during follow-up vs. those with no traumatic knee injury

The analysis of potential differences between skiers who experienced ACL injury during follow-up and those who did not in relation to the biomechanical variables measured at baseline was carried out only for female skiers (Table 5). The number of male skiers who experienced ACL injury in our cohort was too small (*n* = 2) for conducting a meaningful statistical analysis.

**Table 5.** Baseline biomechanical measures of skiers that suffered from a traumatic knee injury/ACL injury during follow-up vs. those with no traumatic injury.

Female skiers only (*n* = 26)*					
	No traumatic knee injury (*n* = 13)*	Traumatic knee injury (*n* = 13)	*p* value	ACL injury (n = 7)	*p* value
Max Knee Flexion Angle at GRF Max	56.3 ± 8.4	46.4 ± 9.0	0.008	45.6 ± 8.2	0.013
Medial knee displacement at GRF max	16.0 ± 7.2	10.6 ± 6.1	0.049	9.4 ± 4.3	0.040

*p* values are based on unpaired sample t tests (*p* < 0.05). The data are presented as the means ± standard deviations.

*Three skiers had to be excluded from this analysis because no valid data from the baseline assessment were available.

Compared with that of female skiers without any traumatic knee injury, the knee flexion angle at the maximal ground reaction force was significantly smaller in female skiers who experienced traumatic knee injury (*p* = 0.008)/ACL injury (*p* = 0.013). The medial knee displacement at the maximal ground reaction force was significantly greater in healthy female skiers than in those who experienced traumatic knee injury (*p* = 0.049) and, likewise, in those who experienced ACL injury (*p* = 0.040).

## Discussion

The main findings of the study were as follows: (1) During the 4-year follow-up, in the cohort of 63 adolescent competitive alpine skiers, 21 skiers experienced at least one traumatic knee injury, 9 of whom experienced ACL injury, with females being significantly more frequently affected. (2) As many as 61.9% of the participating skiers ended their sports careers during the 4 years between 15 and 19 years, 41.0% of whom did so for health-related reasons. (3) At follow-up, 39 skiers presented at least one trauma-related MR imaging abnormality in the knee. (4) At baseline, female skiers who experienced ACL injury during follow-up presented smaller knee flexion angles and greater, although at relatively low magnitudes, medial knee displacement during drop jump landing than non-injured skiers did.

### Traumatic knee injuries during a 4-year follow-up

Alpine skiing is known to be a high-risk sport in terms of traumatic knee injuries and ACL injuries in particular [4–9]. During the follow-up period of 4 years, 33.3% of the adolescent skiers experienced a traumatic knee injury, and 14.3% experienced ACL injury. The latter almost corresponds to the accumulated ACL injury risk for skiers at the elite level, which, according to the literature, ranges between 5% and 15% per season [1,4–6]. Interestingly, in the present study, significantly more female skiers than male skiers experienced ACL injuries. While there is evidence that females are at greater risk for ACL injuries in general, current evidence is inconclusive for competitive alpine skiing [1,37–42]. However, the present findings strongly suggest that during adolescence, female skiers have a greater risk of ACL injuries than their male counterparts do. Moreover, the risk for a secondary ACL injury after an initial ACL injury has been reported to be 6-fold greater within the first 2 years after returning to sport [43]. In the present study, two skiers (22.2%) experienced a re-injury, further highlighting the important role of preventing (or at least delaying) the occurrence of the first ACL injury in adolescent competitive alpine skiers.

### Career status in adolescent alpine skiers

More than 60% of the participating skiers had quit their sports career in competitive alpine skiing during the 4 years between 15 and 19 years. While the most frequently named reasons for quitting were non-health-related, as many as 41.0% of the skiers who left competitive alpine skiing did so for health-related reasons. This further emphasises the important role that health plays in the long-term development and success of aspiring adolescent competitive alpine skiers. According to the Swiss sports system for alpine skiing, the transition from ‘talent’ to ‘elite’ status occurs around the age of 18 years. International races, however, already start at the age of 16 years. In the current study, most athletes quit skiing at the ages of 16 and 17 years before potentially achieving elite status. It is therefore conceivable that many athletes stopped skiing because their prognosis for making it into the ‘elite’ was not promising enough. Previous studies have shown that the fear of failure is relatively high in 15–19-year-old athletes, potentially resulting in high levels of psychological stress or even a predisposition for burnout and ultimately a drop-out from sports careers [44,45]. However, whether and to what extent the described process applies to the skiers of the present study was not analysed.

### Accumulated trauma-related MR imaging abnormalities in the knee

At the follow-up, from a cross-sectional perspective, more than 60% of the 19-year-old skiers included in our study presented at least one trauma-related knee abnormality. In addition to the frequent presence of cartilage lesions and bone marrow edema, persisting abnormalities due to traumatic ACL and PCL injuries were particularly frequent at follow-up assessment (i.e. at the age of 19 years), accounting for 20.6%. In a similar cohort, this ACL and PCL Injury-related abnormality frequency was reported to be 6.5% only at the age of 15 years [18]. In contrast, at the baseline assessment, overuse-related complaints were slightly more prevalent and clinically relevant, whereas at follow-up, only a few skiers had ongoing complaints [18,20]. This shift from overuse- to trauma-related injuries being more frequent from mid- to late adolescence is in line with the literature and has been described to occur after skeletal maturation [46].

### Traumatic injuries and biomechanical risk factors in alpine skiers

Compared with non-injured skiers, female skiers who experienced traumatic knee injury/ACL injury during the 4-year follow-up period presented a decreased knee flexion angle and decreased dynamic knee valgus during drop jump landing at the baseline assessment. The first finding regarding knee flexion is in line with the previously reported hypothesis that jump landing strategies with less flexed knees (i.e. stiff landings) are likely more common in athletes who are prone to ACL injuries [31–33]. Additionally, simulation studies of skiers landing from jumps demonstrated that neuromuscular control patterns characterised by increased hip and knee flexion (i.e. less stiff landings) reduce peak strain acting on the ACL [24,25]. Accordingly, in female skiers, stiff landing strategies may be considered an important but modifiable risk factor that could be targeted through systematic instruction and training.

Regarding the second potential ACL injury risk factor, i.e. increased dynamic knee valgus during jump landings [28–30], we did not observe increased valgus in later injured female skiers but rather the opposite. The medial knee displacement at the maximal ground reaction force was significantly greater (even though at relatively low magnitudes) in healthy female skiers than in those who experienced traumatic knee injury/ACL injury. However, owing to the small magnitude observed, this increased valgus of the knee is not considered to be potentially detrimental in terms of predisposing athletes to ACL injuries. Moreover, Pollard and colleagues reported that in female athletes, knee valgus angles increase with decreasing knee flexion angles during drop jump landing [47], which could be an explanation in view of the stiff landing strategies observed in female skiers who later experienced ACL injuries.

In view of these findings and the high rates of traumatic knee injuries observed in the present study, high-quality jump landing patterns should be an inherent component of prevention strategies in youth and adolescent competitive alpine skiers. In this context, particular attention should be given to soft landings (adequate flexion of the ankle, knee and hip joints in the sagittal plane) and stable leg axes (minimal dynamic knee valgus in the frontal plane). These factors should be addressed in training sessions through corresponding instructions and tailored exercises. Furthermore, as already suggested in an earlier study, education and training on proper jumping techniques should begin at the earliest possible age, as this is critical for athletic development and sustainable health protection [20].

## Limitations

Despite the generally longitudinal design and widely prospective nature of the study, for practicability reasons, the outcomes traumatic knee injuries [yes/no], ACL injuries [yes/no], current sports participation status [active/retired], and if retired reason for dropping out of sports, were assessed by retrospective interviews, at the end of the 4-year follow-up period. This has several limitations that need to be considered when interpreting the study findings. On the one hand, it is possible that the skiers did not remember all the knee injuries they suffered. On the other hand, the reason why they ended their career may have changed in retrospect. Another limitation is that the MR imaging and interviews were not carried out on the same day. Two skiers experienced ACL injury between the time of MR imaging and the time of the interview (approximately 1 month). Although these were not identified during the MR examination of the study, we had to subsequently add them because of the medical diagnosis. Finally, meniscus alterations on MR images can result from different mechanisms, either overuse, such as excessive or adverse loads, or spontaneous healed minor injuries, which do not require medical care. Thus, the term ‘meniscus alterations’, however, might also include overuse-related injuries.

## Conclusion

Approximately one-third of competitive alpine skiers suffer from a traumatic knee injury in the 4 years between 15 and 19 years of age, and every sixth skier from an ACL injury. These rates are higher for female skiers than for male skiers. During the same time, many skiers give up their sports careers during adolescence, with health-related reasons playing an important role. At the age of 19 years, more than 60% of the skiers present trauma-related MR imaging abnormalities in their knees. In female skiers, both sagittal-plane biomechanics and frontal-plane biomechanics differed at baseline between skiers who experienced ACL injury during follow-up and those who did not. Stiff landing strategies may be considered an important but modifiable risk factor that could be targeted through systematic instruction and training.

## Data Availability

The datasets presented in this article are not readily available because their access is restricted to protect the interests of the project partner Swiss-Ski and their athletes. Requests to access the datasets should be directed to joerg.spoerri@balgrist.ch.
